# Selective STAT3 Inhibitor Alantolactone Ameliorates Osteoarthritis *via* Regulating Chondrocyte Autophagy and Cartilage Homeostasis

**DOI:** 10.3389/fphar.2021.730312

**Published:** 2021-09-28

**Authors:** Wenbin Pei, Xiaojian Huang, Bowei Ni, Rui Zhang, Guangyi Niu, Hongbo You

**Affiliations:** ^1^ Department of Orthopedics, Tongji Hospital, Tongji Medical College, Huazhong University of Science and Technology, Wuhan, China; ^2^ Rhode Island School of Design, Providence, RI, United States

**Keywords:** alantolactone, osteoarthritis, autophagy, MMPs, stat3, NF-κB

## Abstract

Osteoarthritis (OA), which is identified by chronic pain, impacts the quality of life. Cartilage degradation and inflammation are the most relevant aspects involved in its development. Signal transducer and activator of transcription 3(STAT3), a member of the STATs protein family, is associated with inflammation. Alantolactone (ALT), a sesquiterpene lactone compound, can selectively suppress the phosphorylation of STAT3. However, the pharmacological effect of ALT on OA is still imprecise. In this study, IL-1β (10 ng/ml) was applied to cartilage chondrocytes, which were treated with different concentrations of Alantolactone for 24 h. The expression of inducible nitric oxide synthase (iNOS), cyclooxygenase-2(COX2), matrix metalloproteinases (MMPs) and thrombospondin motifs-5 (ADAMTS5) were detected by western blot. Protein expression of Collagen Ⅱ was observed by western blot, safranin O staining and immunofluorescence. Manifestation of autophagy related proteins such as autophagy-related gene-5 (ATG5), P62, LC3Ⅱ/Ⅰ and PI3K/AKT/mTOR-related signaling molecules were measured by western blot and autophagic flux monitored by confocal microscopy. Expression of STAT3 and NF-κB-related signaling molecules were evaluated by western blot and immunofluorescence. *In vivo*, 2 mg/kg ALT or equal bulk of vehicle was engaged in the destabilization of medial meniscus (DMM) mouse models by intra-articular injection, the degree of cartilage destruction was classified by Safranin O/Fast green staining. Our findings reported that the enhance of inflammatory factors containing iNOS, COX2, MMPs and ADAMTS5 induced by IL-1β could be ameliorated by ALT. Additionally, the diminish of Collagen Ⅱ and autophagy which was stimulated by IL-1β could be alleviated by ALT. Mechanistically, STAT3, NF-κB and PI3K/AKT/mTOR signal pathways might be involved in the effect of ALT on IL-1β-induced mouse chondrocytes. *In vivo,* ALT protected cartilage in the DMM mouse model. Overall, this study illustrated that ALT attenuated IL-1β-induced inflammatory responses, relieved cartilage degeneration and promoted impaired autophagy *via* restraining of STAT3 and NF-κB signal pathways, implying its auspicious therapeutical effect for OA.

## Introduction

Osteoarthritis (OA) is the most prevailing pattern of arthritis, influencing a large number of people globally (Kloppenburg and Berenbaum, 2020). With the progress of OA, deterioration of articular cartilage, synovial inflammatory responses, and reforging of the subchondral bone become aggravated constantly (Harrell et al., 2019). Patients suffer from clinical symptoms such as pain, structural changes, resulting in disability (Cai et al., 2020). The damage of articular cartilage, which consists of chondrocytes and extracellular matrix (ECM), is an essential event in OA development (Zheng et al., 2021). In addition, pro-inflammatory factors such as iNOS and COX2 are the trigger of modifying inflammatory microenvironment (Cho et al., 2015), leading to the degeneration of ECM, catabolism increasing abnormally and excessive apoptosis (Li et al., 2020). In the advanced stage of osteoarthritis, joint replacement is required generally (Bayliss et al., 2017). On the other hand, in the inchoate stage of osteoarthritis, symptoms and the restriction of joint motion manifest slightly, but the function of the chondrocytes has been altered and gradually affects the joints (Hu et al., 2020). Therefore, it is the effective intervention that should be taken during this period (Taruc-Uy and Lynch, 2013; Ryd et al., 2015). Despite many approaches have been applied to clinical therapy, the valid pharmacology treatment is still deficiency for OA.

Autophagy is a catabolic process that the dysfunctional organelles and harmful cytoplasmic molecules are eliminated. Dysfunctional molecules and organelles are phagocytized by autophagosomes and incorporated to lysosomes for degradation and reclamation during the process of autophagy (Ichimiya et al., 2020). Autophagy flux is the main manifestation of this course. Impaired autophagy is involved in many degenerative diseases such as OA (Luo et al., 2019). Several signal pathways impact autophagic process, one of the most notable is mechanistic target of rapamycin kinase (MTOR). Recently, STAT3 signal pathway was reported that it was also associated with autophagy (Gilardini Montani et al., 2019).

STAT3, a member of the STATs protein family, is involved in cellular proliferation, survival and inflammation (Yu et al., 2009). Each member of the family has analogous molecular configurations, including an amino terminal domain, the coiled-coiled domain (CCD), DNA binding domain, SH2 domain and carboxy-terminal transcriptional activation domain (Furtek et al., 2016). When tyrosine 705(Y705) residues are phosphorylated, a dimer is constituted through the SH2 domain, transfers from the cytoplasm to the nucleus, and binds to the target gene through the DNA binding domain (Lin et al., 2010). Current demonstration which proved that the level of active STAT3 in OA patients’ chondrocytes is superior to the normal group, indicated the vital role of phosphorylated STAT3 in the OA process (Hayashi et al., 2015; Lee et al., 2018). Accordingly, we infer that STAT3 may be the latent target for inflammatory diseases therapy.

ALT, a sesquiterpene lactone, is separated and purified from the *Inula helenium L*. *Inula helenium L* is a glorious nature medicinal plant and has an abundance of bioactivity (Stojakowska et al., 2006; Trendafilova et al., 2010; Cui et al., 2018). Sufficient researches suggested that alantolactone had miscellaneous pharmacologic effects, such as anti-inflammatory, hepatoprotective, antifungal, antibacterial and antitumor effects (Trendafilova et al., 2010). Variously biological activities and beneficial effects of ALT were highlighted in many diseases, including glioblastoma, liver cancer, breast cancer and inflammatory diseases (Khan et al., 2012; Khan et al., 2013; Cui et al., 2018; Dang et al., 2020). Meanwhile, a current investigation confirmed that ALT suppressed the phosphorylation of STAT3 selectively, restraining its transportation between the cytoplasm and nucleus (Maryam et al., 2017). Nevertheless, the regulation of ALT abolishing the sensitization of STAT3 remains indistinct in OA. Based on the above, we analyzed the variation of inflammatory responses, anabolism and autophagy which was impacted by ALT and sought the underlying mechanism in IL-1β-provoked mouse chondrocytes. On the other hand, we evaluated the effects of DMM on mouse cartilage in the experimental OA model and the prognosis of mouse cartilage intervened by ALT in the DMM group.

## Materials and Methods

### Chemicals and Reagents

Alantolactone (HY-N0038) was obtained from MedChem Express (MCE). Recombinant mouse IL-1β cytokine was provided from R&D Systems (501-RL-010, United States). Solarbio supplied safranin O liquor (Beijing, China). Primary antibodies applied in this research were: anti-iNOS, anti-MMP-13 which were acquired from Abcam (Shanghai, China), anti-COX2, anti-LC3Ⅱ/Ⅰ, anti-P-STAT3, anti-P-P65, anti-P-IκB, anti-P/T-mTOR which were purchased from CST (Beverly, MA, United States), anti-STAT3, anti-MMP-1, anti-MMP-3, anti-ATG5, anti-P62, anti-P65, anti-IκB, anti-P/T-PI3K, anti-P/T-AKT, anti-GAPDH which were afforded from Proteintech Group (Wuhan, Hubei, China) and anti-ADAMTS5 which was supplied from Boster Biological Technology (Wuhan, Hubei, China). Secondary antibodies, Cy3 and FITC Conjugated AffiniPure Goat Anti-Rabbit IgG, DAPI staining solution, collagenase type II and trypsin were got from Boster Biological Technology (Wuhan, Hubei, China). HanBio Inc. (Shanghai, China) served the mRFP-GFP-LC3 adenoviral vectors.

### Chondrocyte Isolation and Culture

Chondrocytes were separated from the knee joint cartilage of the 5-day-old C57BL/6J mice which were obtained from The Experimental Animal Center of Tongji Hospital. Shortly, the cartilage was crumbled into miniature patches which were less than 1 mm. Subsequently, utilizing 0.25% trypsin to digest the fragments for 30 min and regurgitating with 0.2% collagenase II for 6 h at 37°C. Afterwards, isolated chondrocytes suspension was centrifuged at 1,500 r/min for 5 min. The sediment of chondrocytes was suspended by the complete medium which contained DMEM/F12 culture medium, 10% fetal bovine serum (FBS, Gibco, NY, United States) and 1% penicillin/streptomycin. Eventually, the chondrocytes, cultured in flasks at 37°C with 5% CO_2_, matured into roughly 90%, passaging and treatment were carried out. The second-generation chondrocytes were selected for succeeding *in vitro* experiments. In addition, all the animal experiments in this study were permitted by the Ethics and Animal Research Committee of Huazhong University of Science and Technology.

### Cell Viability Assay

The toxic effect of ALT on mice articular chondrocytes was evaluated by the CCK8 kit. Chondrocytes, seeded into 96-well plates at the density of 5 × 10^3^ per well, was stimulated with different concentrations of ALT with or without IL-1β (10 ng/ml) for 24 h. Thenceforth, each well was added in mixture which contained 100 μl DMEM/F12 culture medium and 10 μl CCK-8 solution. An hour later, the viability of chondrocytes was detected by the microplate reader (Bio-Rad, Richmond, CA, United States) at the wavelength of 450 nm.

### Cellular Safranin O Staining

The relative content of proteoglycan, according to the safranin O staining brightness, could be observed intuitively by safranin O. Concisely, chondrocytes, seeded into a 6-well plate, were motivated by IL-1β (10 ng/ml) with or without ALT (10 μM). The medium was renewed every 3 days until the seventh day. Subsequently, each well was fixed in 4% paraformaldehyde (PA) for 30 min and washed by PBS after the fixation. At last, the safranin O solution was utilized to the steady chondrocytes for 30 min, which should be removed and washed by PBS three times for 5 min each after incubation. To estimate the color variation of each well, pictures of varying red staining intensities were captured by a microscope (Evos Fl Auto, Life Technologies, United States).

### Immunofluorescence Staining

24 well plates were prepared for Collagen II, STAT3 and P65 staining, which were seeded into 2 × 10^4^ per well. Chondrocytes, stimulated by IL-1β (10 ng/ml) with or without ALT (10 μM), were applied to be fixed in 4% PA for 15 min. After being fixed, the cells were covered with 0.2% Triton X-100 for 10 min and blocked with 1% BSA for 30 min at normal temperature, subsequently, of which the antigens were combined with primary antibodies against Collagen II, STAT3 and P65 at 4°C overnight. The chondrocytes, washed by PBS, were incubated with Cy3 Conjugated AffiniPure Goat Anti-Rabbit secondary antibody for 1 h at room temperature darkling. Finally, the nucleuses of chondrocytes were dyed by DAPI for 10 min. Photographs were observed and acquired by a fluorescence microscope.

### mRFP-GFP-LC3 Adenovirus Infection and Confocal Microscopy

The multiplicity of infection (MOI), determined by preliminary experiments, was 20 μl. Chondrocytes were infected with adenoviral vectors at approximately 50% confluence. After 12 h of infection, the cells were cultured in DMEM/F12 medium for 48 h. Subsequently, chondrocytes were treated by IL-1β with or without ALT (10 μM) and fixed with 4% paraformaldehyde after 24 h. A Nikon C2+ laser scanning confocal microscope (Nikon America Inc., Melville, NY) was used to record the autophagy flux.

### Western Blotting

According to the manufacturer’s instructions, cytoplasmic and nuclear proteins were extracted from Isolated chondrocytes by using the nuclear and cytoplasmic protein extraction kit (Beyotime, Beijing, China). Cells, stimulated by IL-1β (10 ng/ml) with or without ALT (2.5, 5, and 10 μM), were lysed with RIPA Lysis Buffer containing 1% of protease and phosphatase inhibitors for 15 min on ice. Collecting the lysed products and centrifugating at 12,000 r/min for 30 min at 4°C. The gathered supernatant concentration was measured by a BCA kit (Boster, China) and using microplate reader (Bio-Rad, Richmond, CA, United States) at the wavelength of 562 nm. 12% SDS-PAGE was applied to separate Protein samples and markers, which were transferred to 0.45 nm PVDF membranes (Millipore, United States) subsequently. Primary antibodies were utilized to combined with the corresponding proteins in the membranes which were blocked with 5% BSA for 1 h. After the incubation at 4°C overnight, the membranes were washed three times with Tris Buffered Saline Tween (TBST). Afterwards, secondary antibodies were applied to the membranes for 1 h at room temperature, followed by washing in TBST three times. The purpose protein was evaluated by Supersensitive ECL kit (Boster, Wuhan) and a Bio-Rad scanner (Bio-Rad, United States), and ImageJ software was used to analyze the gray level of each protein band. Western blotting results were repeated at least three times.

### Animal Experiments

The Experimental Animal Centre of Tongji Hospital provided twenty-four 8-week-old male C57BL/6J mice which were feeding in the SPF animal laboratory. The establishment of mouse OA model was based on the surgical procedure that medial meniscus was destabilized. Briefly, the destabilization of medial meniscus was operated on the left knee of mice which were stupefied by intraperitoneal injection of 9% chloral hydrate and after the operation, the skin was seamed. Three groups were divided at random (*n* = 8 per group), the sham group, the DMM group and the DMM + ALT group. ALT has poor solubility in physiological saline but soluble in DMSO, so we choose DMSO as the solvent. Before the animal experiment, add the ALT stock solution to 20% SBE-β-CD in saline at a ratio of 1:9 and mix well. ALT (2 mg/kg) dissolved in 10 μl of a solution was injected in the joint cavity of DMM + ALT group mice twice a week for 8 consecutive weeks. The 10 μl of the vehicle was injected to sham group and the DMM group. After 8 weeks, all the mice were sacrificed by neck snap and each left knee was separated for further analysis. All the animal experiments in this study were admitted by The Institutional Animal Care and Use Committee of Tongji Hospital (Wuhan, China).

### Histological and Immunohistochemistry Assessment

Using 4% paraformaldehyde to fix the gathered joints for 24 h after that the treated joints were decalcified in 10% EDTA for a month. After paraffin embedding, samples were sliced to 5 μm sections and then stained with Safranin O/Fast Green. The severity of cartilage damage was assessed by the Osteoarthritis Research Society International (OARSI) guidelines. After deparaffinized, the sections were blocked with 5% BSA for 1 h. Then, samples were incubated with primary antibodies against Collagen II and MMP13 at 4°C overnight. After incubated with secondary antibodies, images were observed and acquired by a microscope.

### Statistical Analysis

GraphPad Prism v.8.4.0 software was used to analyze experimental data. The results were shown as the mean ± SD. Significant differences were determined applying one-way analysis of variance (ANOVA) followed by Tukey’s test. *p* < 0.05 was recognized significant. All data were obtained through at least three independent repeated experiments.

## Result

### The Identification of Mouse Chondrocytes

**FIGURE 1:**
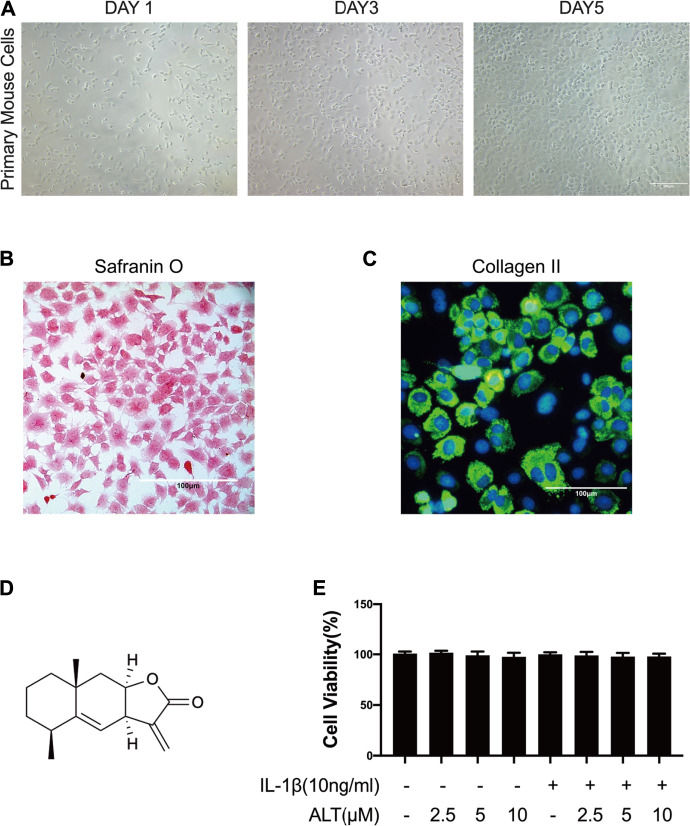
The identification of mouse chondrocytes and ALT did not impact the viability of mouse chondrocytes. **(A)** Phase-contrast micrographs of primary mouse cells after isolation day 1, 3, and 5. **(B)** Safranin O staining of primary mouse cells. **(C)** Collagen II immunofluorescence staining of primary mouse cells. Cells were treated by ALT (2.5, 5, and 10 μM) in the presence or absence of IL-1β (10 ng/ml) for 24 h. **(D)** Chemical structure of ALT. **(E)** Cell viability was determined by CCK-8 assay.

The proteoglycan and Collagen II are main components of the extracellular matrix in chondrocytes. Evidences showed that the extracellular matrix produced by mouse chondrocytes could not only confer flexibility to the joint surface, but also mineralize. These differences were due to the expression of proteoglycan and COL2 in chondrocytes (Gosset et al., 2008). Therefore, in this study, phase-contrast microscopy, safranin O staining and the immunofluorescence of Collagen II were used to identify the primary mouse cells we isolated. As shown in Figures 1A–C, primary mouse cells exhibited the typical chondrocyte morphology, with a rounded or polygonal shape and granular cytoplasm under the phase-contrast microscopy. The proteoglycans were stained with red by safranin O and the Collagen II was stained with green fluorescence in the cytoplasm.

### ALT Did Not Impact the Viability of Mouse Chondrocytes

CCK8 kit was used to evaluate the cytotoxic effect of ALT on mouse articular chondrocytes. Cells were stimulated by ALT (2.5, 5, and 10 μM) in the presence or absence of IL-1β (10 ng/ml) for 24 h. Results in Figure 1E, the cell viability was not impacted by various concentrations of ALT. Therefore, 2.5, 5, and 10 μM ALT were utilized for subsequent experiments.

### ALT Moderated IL-1β-Induced iNOS and COX2 Expression in Mouse Chondrocytes

**FIGURE 2:**
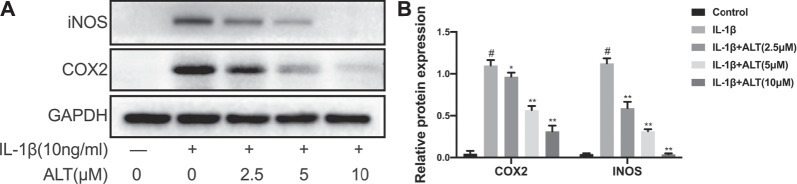
ALT moderated IL-1β-induced iNOS and COX2 expression in mouse chondrocytes. Cells were stimulated by IL-1β (10 ng/ml) in the presence or absence of ALT (2.5, 5, and 10 μM) for 24 h. **(A)** Expression of iNOS and COX2 were detected by Western blot. **(B)** Relative protein expression was qualified by ImageJ software, GAPDH was served as the loading control (*n* = 3). #*p* < 0.05 vs. control group; *p < 0.05 vs. IL-1β group; **p < 0.01 vs. IL-1β group.

COX2 and iNOS, the major pro-inflammatory cytokines in the development of OA, are abnormal raising (Stefik et al., 2021). In this study, chondrocytes were stimulated by ALT in the presence or absence of IL-1β (10 ng/ml) for 24 h. As shown in Figure 2, iNOS and COX2 were enhanced remarkably in IL-1β-induced chondrocytes. However, ALT could significantly moderate the excess expression of COX2 and iNOS in dose-dependently.

### ALT Alleviated Excess MMPs and ADAMTS5 Expression in IL-1β-Stimulated Mouse Chondrocytes

**FIGURE 3:**
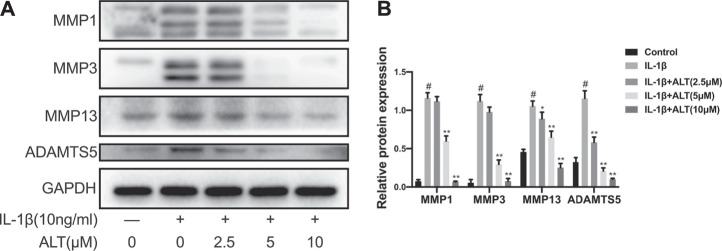
ALT alleviated excess MMPs and ADAMTS5 expression in IL-1β-stimulated mouse chondrocytes. Chondrocytes were exposed to ALT (2.5, 5, and 10 μM) with or without IL-1β (10 ng/ml) for 24 h. **(A)** Western blot was employed to determine the expression of MMP1, MMP3, MMP13, and ADAMTS5. **(B)** Relative protein expression was qualified by ImageJ software, GAPDH was used as the internal control (*n* = 3). #*p* < 0.05 vs. control group; *p < 0.05 vs. IL-1β group; **p < 0.01 vs. IL-1β group.

It is MMPs and ADAMTS5 that are the main catabolic enzymes in the ECM and the expression levels are augmenting during the development of OA (Zheng et al., 2021). Therefore, we evaluated the effects of ALT on the protein expression levels of MMP-1, MMP-3, MMP-13 and ADAMTS5. As shown in Figures 3A,B, IL-1β augmenting the expression levels of MMPs and ADAMTS5 anomalously. However, ALT in high concentrations (5 and 10 μM) mitigated the protein expression of MMPs and ADAMTS5 stimulated by IL-1β. ALT in low concentration (2.5 μM) decreased the expression of MMPs that was no statistical difference.

### ALT Attenuated the Degeneration of Collagen II Induced by IL-1β in Mouse Chondrocytes

**FIGURE 4:**
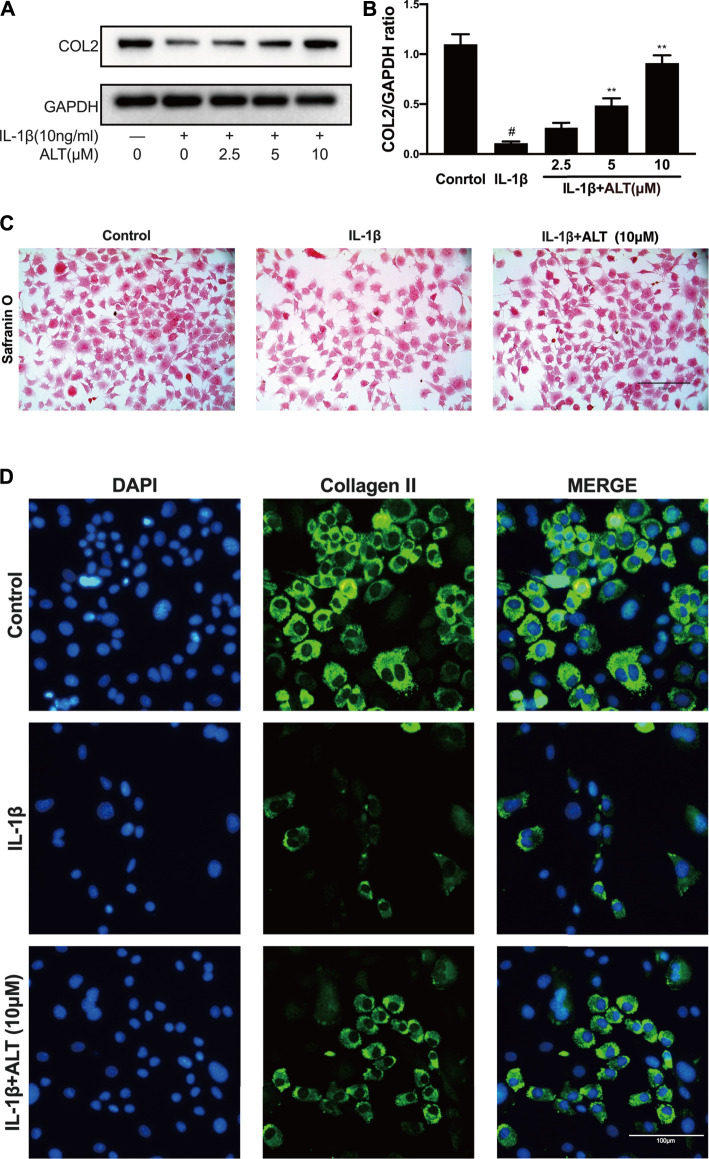
ALT attenuated the degeneration of Collagen II induced by IL-1β in mouse chondrocytes. **(A)** Western blotting result of Collagen II. **(B)** Quantification analysis of western blotting results, GAPDH was regarded as an internal control (*n* = 3). **(C)** Safranin O staining for proteoglycans deposition in each group after a 7-days incubation (scale bar 100 μm). **(D)** Collagen II expression was determined by immunofluorescence staining (scale bar 100 μm). #*p* < 0.05 vs. control group; *p < 0.05 vs. IL-1β group; **p < 0.01 vs. IL-1β group.

As one of the main components of extracellular matrix, Collagen II impacts on the anabolism of chondrocytes. Therefore, we measured the protein level of Collagen II induced by IL-1β in the presence or absence of ALT, and stained mouse chondrocytes with Safranin O. As shown in Figures 4A,B, after stimulating mouse chondrocytes with IL-1β for 24 h, the protein level of Collagen II was decreased, while ALT could relieve the restraint of Collagen II caused by IL-1β. 10 μM concentration of ALT which could attenuate the degeneration prominently was selected to apply in safranin O staining and Immunofluorescence staining. Chondrocytes stained with Safranin O (Figure 4C) showed that compared with control group, the density and redness of chondrocytes were diminished in IL-1β group. However, in ALT treatment group, the density and redness of chondrocytes were increasing compared with IL-1β group. Fluorescent result showed Collagen II (green light) was reduced in IL-1β-induced chondrocytes and after the treatment of ALT, Collagen II (green light) was augmented compared with the IL-1β group (Figure 4D).

### ALT Mitigated Impaired Autophagy Induced by IL-1β in Mouse Chondrocytes

**FIGURE 5:**
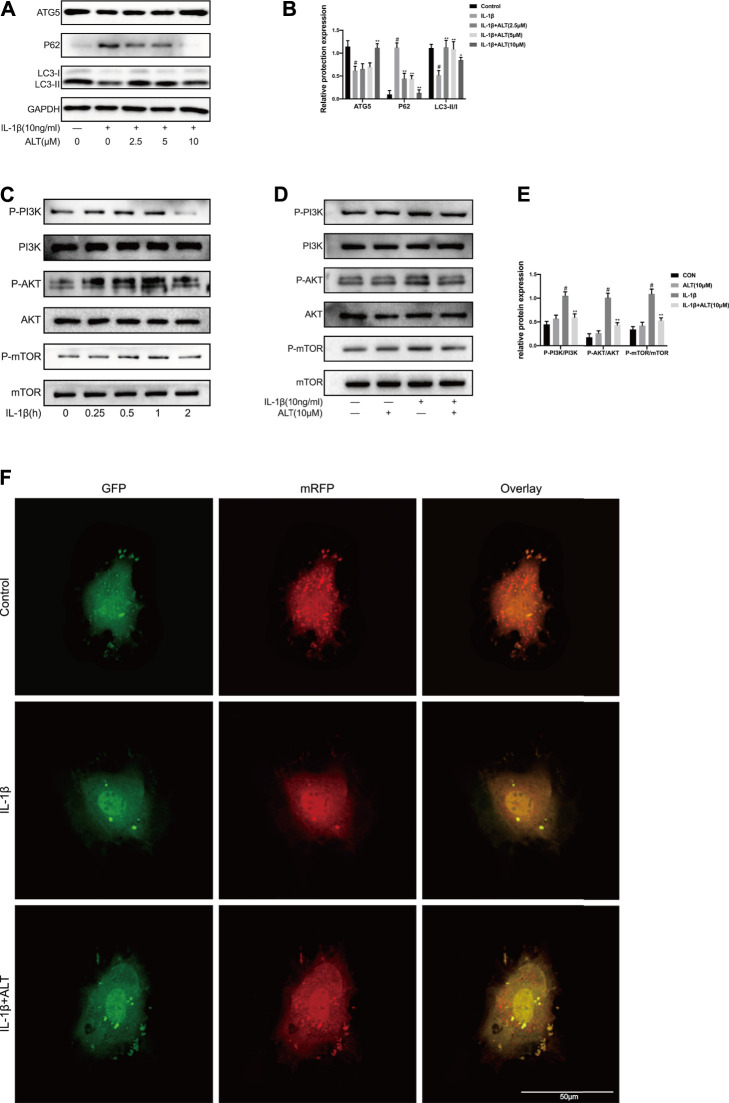
ALT mitigated impaired autophagy induced by IL-1β in mouse chondrocytes. Cells were stimulated by IL-1β (10 ng/ml) with or without ALT (2.5, 5, and 10 μM) for 24 h. **(A)** Western blot was employed to detect the expression of ATG5, P62 and LC3Ⅱ/Ⅰ. **(B)** Relative protein expression was qualified by ImageJ software, GAPDH was used as the internal control, respectively (*n* = 3). **(C)** Mouse chondrocytes were exposed to IL-1β (10 ng/ml) at different time points (0, 0.25, 0.5, 1, and 2 h). PI3K/AKT/mTOR-related signaling molecules was determined by Western blot. **(D)** Cells were exposed to ALT (10 μM) with or without IL-1β (10 ng/ml) for 1 h. Protein levels of P-PI3K, PI3K, P-AKT, AKT, P-mTOR and mTOR were detected by Western blot. **(E)** Relative protein expression was qualified by ImageJ software, PI3K, AKT and mTOR were used as the loading control, respectively (*n* = 3) **(F)** Chondrocytes were transfected with the mRFP-GFP-LC3 adenovirus for 24 h and treated by IL-1β (10 ng/ml) with or without ALT (10 μM) for 24 h. Autophagosomes were represented by yellow puncta and autolysosomes by red puncta in merged images. #*p* < 0.05 vs. control group; *p < 0.05 vs. IL-1β group; **p < 0.01 vs. IL-1β group.

P62 and LC3Ⅱ/Ⅰ are regarded as the mark of autophagy, the expression levels of which reflect the tendency of autophagy (Lorzadeh et al., 2021). To investigate the influence of ALT on autophagy, chondrocytes were stimulated by IL-1β with or without ALT for 24 h. As shown in Figures 5A,B, the up-regulation of P62 and the degradation of LC3Ⅱ/Ⅰ and ATG5 in autophagy was induced by IL-1β. After treatment with ALT, the augment of P62 and the lessen of LC3Ⅱ/Ⅰ and ATG5 were mitigated. The result of western blot was in accord with mRFP-GFP-LC3 adenovirus infection (Figure 5F). The red spots are autolysosomes (mRFP) and the yellow spots are autophagosomes (RFP + GFP). Compared with the control group, the red intensity induced by IL-1β indicated the formation of autolysosome was reduced. The trend was alleviated by ALT treatment, with increased autophagosome transformed to autolysosomes. To explore the mechanism, chondrocytes were stimulated with IL-1β (10 ng/ml) at different time points (0, 0.25, 0.5, 1, and 2 h). As shown in Figure 5C, PI3K/AKT/mTOR pathway was activated at 1 h and the timing was utilized in subsequent experiments. To investigate the effect of ALT on PI3K/AKT/mTOR pathway, mouse chondrocytes were treated by ALT (10 μM) with or without IL-1β for 1 h. As shown in Figure 5D, the expressions of P-PI3K, P-AKT and P-mTOR were upregulated by IL-1β. After ALT treatment, the trend could be partly attenuated.

### ALT Restrained the Phosphorylation of STAT3 Induced by IL-1β in Mouse Chondrocytes

**FIGURE 6:**
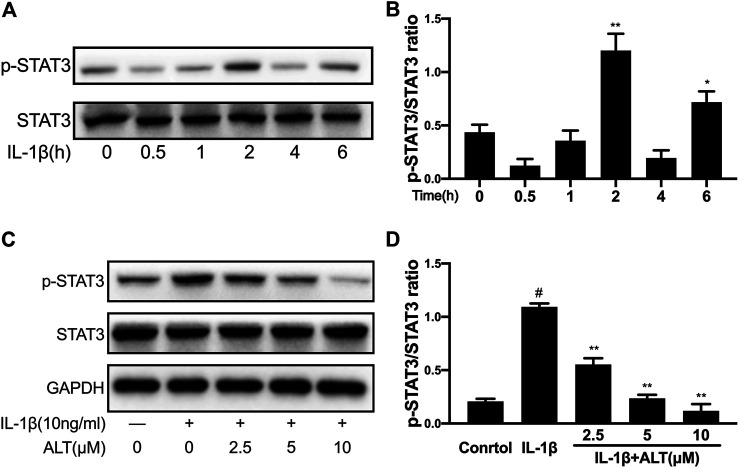
ALT restrained the phosphorylation of STAT3 induced by IL-1β in mouse chondrocytes. **(A)** Mouse chondrocytes were exposed to IL-1β (10 ng/ml) at different time points (0, 0.5, 1, 2, 4, and 6 h). Phosphorylation of STAT3 was determined by Western blot. **(B)** Relative protein expression was qualified by ImageJ software, STAT3 was used as the internal control (*n* = 3). **(C)** Cells were treated by ALT (2.5, 5, and 10 μM) with or without IL-1β (10 ng/ml) for 2 h. Phosphorylation of STAT3 was determined by Western blot. **(D)** Relative protein expression was qualified by ImageJ software, STAT3 was used as the loading control (*n* = 3). #*p* < 0.05 vs. control group; *p < 0.05 vs. IL-1β group; **p < 0.01 vs. IL-1β group.

To explore optimum timing when the phosphorylation of STAT3 was stimulated by IL-1β, the mouse chondrocytes were stimulated with IL-1β (10 ng/ml) at different time points (0, 0.5, 1, 2, 4, and 6 h). As shown in Figures 6A,B, STAT3 was activated obviously after 2 h of IL-1 stimulation and the timing was used in subsequent experiments. To investigate the effect of ALT on IL-1β-induced phosphorylation of STAT3, an experiment was designed to treat mouse chondrocytes by ALT with or without IL-1β. As shown in Figures 6C,D, which was verified by previous experiments, IL-1β activated the phosphorylation of STAT3. After the treatment with ALT, the phosphorylation of STAT3 induced by IL-1β was restrained in dose-dependently.

### ALT Suppressed IL-1β-Induced STAT3 Translocation in Mouse Chondrocytes

**FIGURE 7:**
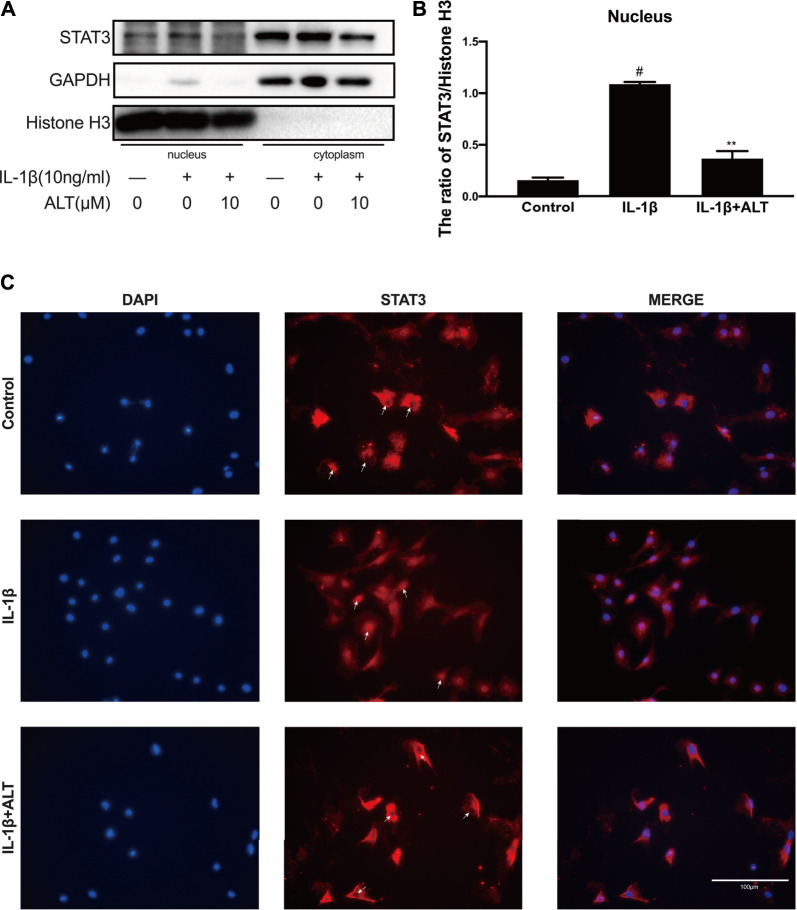
ALT suppressed IL-1β-induced STAT3 translocation in mouse chondrocytes. Mouse chondrocytes were treated with or without ALT (10 μM) and stimulated with IL-1β (10 ng/ml) for 2 h. **(A)** Protein level of STAT3 in nucleus was detected by Western blot. **(B)** Relative protein expression was qualified by ImageJ software, Histone H3 was used as the loading control (*n* = 3). **(C)** STAT3 translocation was observed by Immunofluorescence (scale bar 100 μm). #*p* < 0.05 vs. control group; *p < 0.05 vs. IL-1β group; **p < 0.01 vs. IL-1β group.

Phosphorylated STAT3 enters the nucleus from the cytoplasm in the form of a dimer, and works with the combination of it and the target gene (Schaefer et al., 1997). In order to examine the effect of ALT on STAT3 nuclear transport, mouse chondrocytes were treated with or without ALT (10 μM) and stimulated with IL-1β. Nuclear protein and plasma protein were separated and extracted with Nuclear and Cytoplasmic Protein Extraction Kit. As shown in Figures 7A,B, the level of STAT3 in the nucleus was increased with IL-1β-stimulated in the chondrocytes. After ALT treatment, the level of IL-1β-induced STAT3 in the nuclear was reduced, that was confirmed by immunofluorescence. As shown in Figure 7C, STAT3 (red light label), stimulated with IL-1β in the chondrocytes, mainly gathered in the nucleus. After treated with ALT, STAT3 (red light label) displayed in the cytoplasm and there was no redness signal in the nucleus.

### ALT Relieved IL-1β-Induced NF-κB Signaling Pathway Activation in Mouse Chondrocytes

**FIGURE 8:**
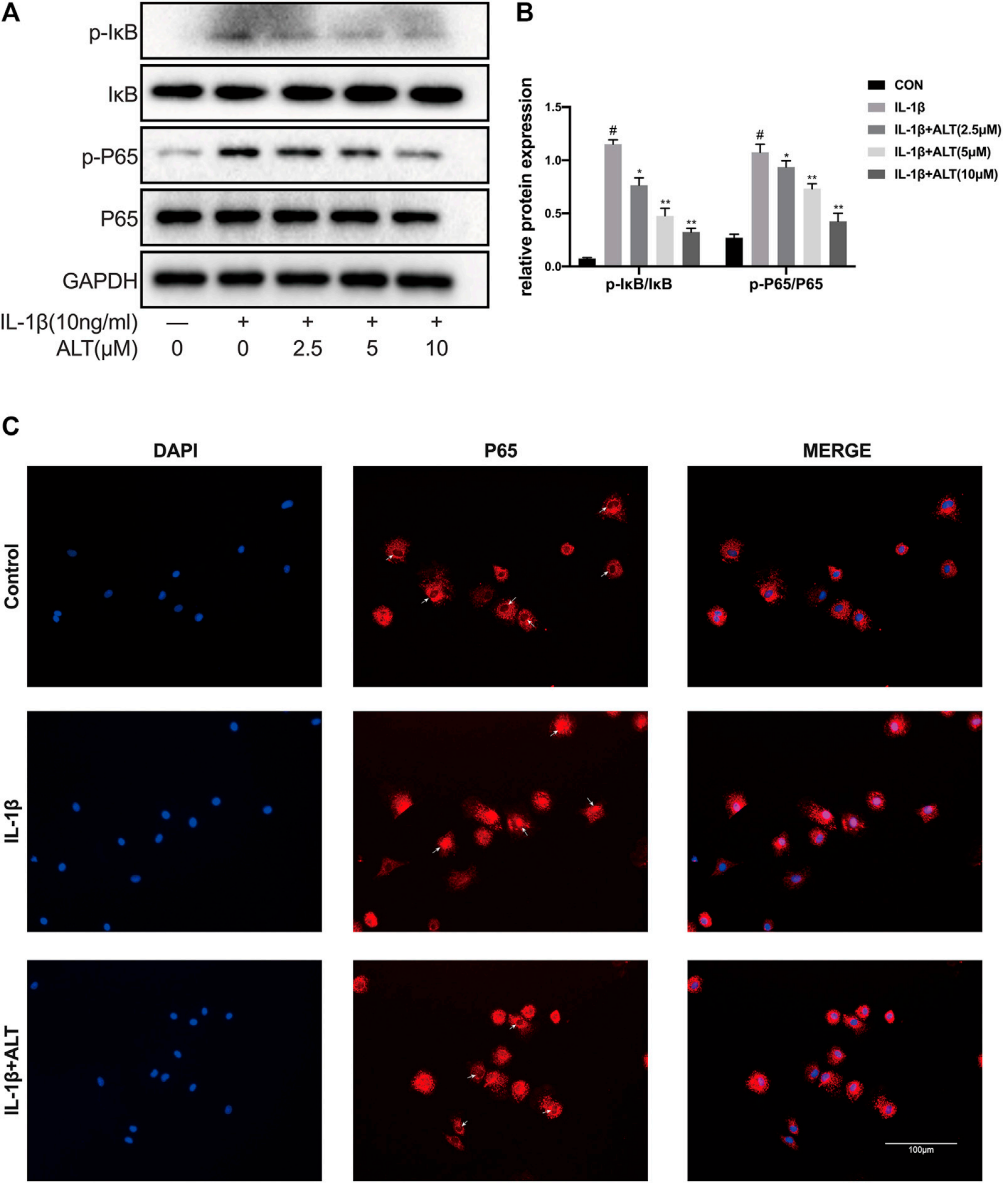
ALT relieved IL-1β-induced NF-κB signaling pathway activation in mouse chondrocytes. Cells were exposed to ALT (10 μM) with or without IL-1β (10 ng/ml) for 30 min. **(A)** Protein levels of p- IκB, IκB, p-p65, p65 were detected by Western blot. **(B)** Relative protein expression was qualified by ImageJ software, IκB and p65 were used as the loading control, respectively (*n* = 3). **(C)** p65 translocation was observed by Immunofluorescence (scale bar 100 μm). #*p* < 0.05 vs. control group; *p < 0.05 vs. IL-1β group; **p < 0.01 vs. IL-1β group.

STAT3 and NF-κB have been demonstrated to cooperate in cell growth by interacting at different levels of their activating pathways (Zhou et al., 2019). Therefore, we explored the effect of ALT on the NF-κB signaling pathway. Chondrocytes were stimulated by IL-1β with or without ALT for 30 min, the results were shown in Figures 8A,B, that the expressions of P-IκB and P-P65 were upregulated by IL-1β. After ALT treatment, the trend could be partly relieved in a dose–dependent manner. The fluorescent result was consistent with the western blot that ALT (10 μM) could significantly restrict nuclear translocation of P65 induced by IL-1β in chondrocytes (Figure 8C).

### ALT Attenuated the Vitiation of Cartilage in the Mouse OA Model

**FIGURE 9:**
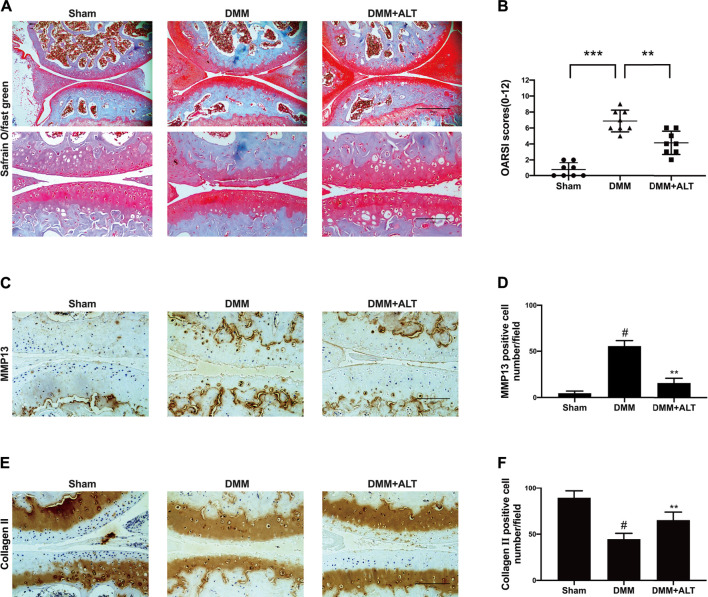
ALT attenuated the vitiation of cartilage on the mouse OA model. **(A)** Microscopic photos of Safranin-O-Fast green stained mouse knee joint sections of three groups (scale bar 200 and 100 μm). **(B)** The OARSI scores of each group. **p < 0.01; ***p < 0.001 **(C)** Immunohistochemistry staining of MMP13. **(D)** Number of MMP13 positive cells per field under 100-time magnification. **(E)** Immunohistochemistry staining of Collagen II. **(F)** Number of Collagen II positive cells per field under 100-time magnification. #*p* < 0.05 vs. sham group; **p < 0.01 vs. DMM group.

According to the previously mentioned methods, we established a mouse OA model. After the surgery and interventions, left knees of three groups were gathered. During the period, infections or other postoperative complications was not discovered in mice. Compared with the severe cartilage surface erosion in DMM group, samples from the sham group showed the normal structure with lippy cartilage surface. Contrasted with the DMM group, Intra-articular injection of ALT (2 mg/kg) could attenuate cartilage destruction (Figures 9A,B). Immunohistochemistry staining of MMP13 and Collagen II were utilized to evaluate the catabolism and anabolism of chondrocyte. Compared with control group, the number of MMP13 positive cell increased in DMM group and intra-articular injection of ALT (2 mg/kg) decreased the MMP13 positive cell number (Figures 9C,D). The number of Collagen II positive cell reduced in DMM group and after the treatment of ALT, the number of Collagen II positive cell increased in DMM + ALT group (Figures 9E,F).

## Discussion

**FIGURE 10:**
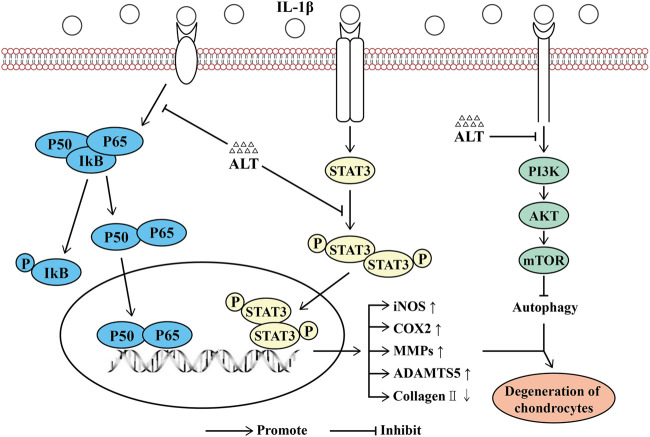
Schematic diagram of the effect of ALT on cartilage degeneration. IL-1β induces the expression of pro-inflammatory factors, including iNOS, COX2, MMPs and ADAMTS5. IL-1β stimulates the degradation of Collagen II and the impairment of autophagy. Furthermore, IL-1β functions by activating the PI3K/AKT/mTOR, NF-κB signaling pathways, phosphorylating and translocating STAT3. As shown in the figure, the levels of P-PI3K, P-AKT, P-mTOR, P-IKB, P-P65, P-STAT3 and the translocation of P65 and STAT3 are increasing. However, ALT can attenuate these effects.

Osteoarthritis is a kind of widespread joint degenerative disease, which is related to many factors, such as age, gender, inflammation, stress, and obesity (Loeser, 2009; Osteras et al., 2019). In recent years, the treatment strategies for osteoarthritis are mainly divided into non-pharmacological, pharmacological and surgical operation treatment (Bijlsma et al., 2011). For early-stage patients, non-surgical treatments are generally considered effective, such as weight loss and exercise (Hunter and Bierma-Zeinstra, 2019), data also showed that exercise therapy significantly reduced pain and improved the quality of life (Fransen et al., 2015). There was evidence that in the short term (less than 12 months), weight loss and exercise therapy could moderately improve pain and physical function (Hall et al., 2019). In terms of pharmacological treatment, paracetamol and non-steroidal anti-inflammatory drugs are the first-line drugs for the treatment of osteoarthritis pain (Bannuru et al., 2019; Hunter and Bierma-Zeinstra, 2019). For advanced patients, surgical treatment of joint replacement is widely used. Comprehensive comparison of these three treatment methods shows that pharmacological treatment could be more advantageous, economical and highly medically compliant, which has obvious curative effects. Non-steroidal anti-inflammatory drugs (NSAIDs) and COX2-inhibitors as first-line drugs for the treatment of osteoarthritis can alleviate the pain symptoms of OA (Cho et al., 2015). However, the drugs in clinical for OA are focused on relieving symptoms and with the development of OA, the damage of cartilage will be severe. It is imperative to find effective drugs for the treatment of OA. ALT, a sesquiterpene lactone, is mainly extracted from the roots of Inula helenium L, which has potential anti-inflammatory, anti-bacterial, anti-fungal, anti-tumor effects and selectively inhibits STAT3 (Stojakowska et al., 2006). Some data showed that ALT had an anti-inflammatory effect in RAW264.7 cells stimulated by LPS(Chun et al., 2012), in other diseases, such as breast cancer (Cui et al., 2018), liver cancer (Khan et al., 2013), and glioma (Khan et al., 2012), ALT also shown its potential therapeutic effects. In this study, we were the first to report the effect of ALT on IL-1β-induced negative effects by suppression of STAT3 and NF-κB signaling pathways in mouse chondrocytes (Figure 10).

IL-1β, considered to be a pro-inflammatory factor that plays a critical role in OA. In the synovium, chondrocytes, and subchondral bone of patients with OA, the level of IL-1β increased, which resulted in the decrease of extracellular matrix (ECM) and the enhancement of matrix metalloproteinases (MMP1, MMP3, MMP13) and ADAMTS5 (Kapoor et al., 2011). IL-1β could significantly restrain the synthesis of collagen II and enhanced of inflammatory factors, such as iNOS and COX2. Previous studies reported that the stimulation of IL-1β (10 ng/ml) for 24 h could induce inflammation in chondrocytes (Huang et al., 2019; Hou et al., 2021). In this study, IL-1β (10 ng/ml) induced inflammatory responses in mouse chondrocytes indeed. ALT could apparently attenuate the negative effects of chondrocytes induced by IL-1β. *In vivo*, we established a mouse OA model through destabilization of the medial meniscus (DMM) to evaluate the protective effect of ALT on cartilage degradation. Combining the data of *in vivo* and *in vitro*, ALT could alleviate inflammatory responses and cartilage degeneration induced by IL-1β, which might be an effective treatment for OA. Autophagy, a highly conservative process, which is associated with cellular homeostasis and nutrient regulation. Impaired organelles and deteriorative proteins which cannot be digested by the proteasome are transported to the lysosomal for degradation. Autophagy is divided into three types: macroautophagy, microautophagy and chaperone-mediated autophagy (Ichimiya et al., 2020). It was reported that autophagy had both advantageous and disadvantageous influences in many diseases. It was investigated recently that in the myocardial ischemia–reperfusion (I/R) injury, autophagy enhanced the inflammatory responses. In other inflammatory diseases, autophagy is regarded as a kind of beneficial factor, such as inflammatory bowel disease, chronic obstructive pulmonary disease and osteoarthritis (Xue et al., 2017; Racanelli et al., 2018; Jin and Zhang, 2020). Several signal pathways are involved in the regulation of autophagy, such as PI3K/AKT/mTOR signal pathway and AMPK signal pathway (Kim et al., 2011). Furthermore, the crosstalk with other signal pathways, including STAT3 impacts the process of autophagy. Some studies reported that STAT3, transported to the nucleus and bound to the DNA, suppressed the target genes of autophagy, such as Bcl-2, BECN1 and PIK3C3(You et al., 2015; Liu et al., 2017). In this study, the data showed that ALT relieved the reduction of autophagy flux and autophagosome formation induced by IL-1β *via* suppressing the PI3K/AKT/mTOR signal pathway in mouse chondrocytes. While 3-methyladenine (3-MA) is generally considered as an inhibitor of autophagy (Zhou et al., 2021), the effect of ALT with 3-MA on IL-1β-induced impaired autophagy is not explored in this study.

JAK/STATs signaling pathway plays a vital role in inflammation, cell proliferation, and apoptosis, which could be activated by many cytokines, like growth factors, and interferons. It was investigated that inhibiting the activation of the JAK/STAT3 signaling pathway could mitigate the cartilage degradation induced by IL-1β (Liu et al., 2018). After the tyrosine residues of STAT3 are phosphorylated in the cytoplasm, they were transported to the nucleus in the form of dimers and bind to specific DNA sequences to function (Bharadwaj et al., 2020). Although some studies shown that phosphorylation of STAT3 was not a compulsory factor to lead to its nuclear transport, non-phosphorylated STAT3 could not bind to specific STAT sites on DNA (Liu et al., 2005). From our experimental results, IL-1β could significantly rise the phosphorylation of STAT3 in mouse chondrocytes and enhance its transport to the nucleus. However, this process could be suppressed by ALT which was identified with immunofluorescence results. Interestingly, we found that the phosphorylation level of STAT3 increased at 2 and 6 h, and decreased at 4 h. This might be related to the activation of the upstream protein tyrosine phosphatase (PTP) including SHP-1, SHP-2 and PTEN (Yu et al., 2010; Nair et al., 2012). Experiments shown that the inhibitory effect of ALT on the phosphorylation of STAT3 required the participation of PTP, but ALT did not affect the activity of PTP (Chun et al., 2015). Therefore, we suspected that the phosphorylation of STAT3 increased rapidly after IL-1β stimulation at the early stage. With increased phosphorylation level of STAT3, the feedback between PTP and STAT3 regulated the phosphorylation level of STAT3 to reduce its phosphorylation. Subsequently, the constant stimulation of IL-1β unbalanced this feedback loop and rose the phosphorylation level of STAT3 again. To consummate the research, upstream of STAT3, such as PTP, JAK, should be evaluated and si-STAT3 should be utilized in follow-up study to acquire the accurate conclusion by comparing the results.

Transcription factor NF-κB plays an essential character in inflammatory responses as well, which was combined with the inhibitory κB protein (IκB) in the cytoplasm to maintain non-activated state. Stimulated by cytokines such as IL-1β, IκB could be phosphorylated and degraded by ubiquitination, which in turn promoted phosphorylation of P65 and transported it into the nucleus to regulate the expression of the target gene (Roman-Blas and Jimenez, 2006). NF-κB and STAT3, two transcription factors involved in inflammation, can crosstalk at multiple levels. P65, the member of NF-κB, interacts with STAT3 and bind at gene promoters to induce target genes expression (Fan et al., 2013). In addition to participating in inflammatory responses, NF-κB is also involved in the regulation of autophagy, which can inhibit autophagy by increasing the expression of autophagy repressors, such as Bcl-2 family and PTEN/mTOR (Verzella et al., 2020). Some studies have showed that the impaired autophagy could be alleviated by the inhibit or knockdown of NF-κB (Chen et al., 2020). Our data indicated that IL-1β could obviously increase the phosphorylation of IκB and P65, which were suppressed by ALT in dose-dependently, suggesting the chondroprotective effect of ALT might by the suppression of NF-κB pathway. However, the connection of STAT3 and NF-κB signal pathway was indistinct, which need to be explored in the future.

In conclusion, our *in vitro* findings revealed that ALT could attenuate the inflammatory responses, cartilage degeneration and impaired autophagy induced by IL-1β by suppressing the nuclear transport of STAT3 and the NF-κB signal pathway. *In vivo* experiments suggested that the damage of cartilage and the release of inflammatory factors such as MMP13 in DMM group, could be alleviated after the treatment of ALT. Our data showed the therapeutic potential of ALT to the experimental OA.

## Data Availability

The raw data supporting the conclusion of this article will be made available by the authors, without undue reservation.
